# The Role of Digital Primary Care on Cardiovascular Health in a Changing Climate

**DOI:** 10.1055/s-0046-1816593

**Published:** 2026-11-03

**Authors:** Jitendra Jonnagaddala, Debasish Kar, Craig Kuziemsky, Karen Tu, Sabrina Kay Wye Wong, Myron Godinho, Anthony Paulo Sunjaya, María Sofía Cuba Fuentes, Javier Silva-Valencia, Alvin B Marcelo, Simon de Lusignan, Siaw-Teng Liaw

**Affiliations:** 1 School of Population Health, UNSW SydneyKensingtonAustralia; 2 Community and Primary Care Research Group, University of PlymouthPlymouthUK; 3 MacEwan UniversityEdmontonAlbertaCanada; 4 Department of Family and Community Medicine, University of TorontoTorontoONCanada; 5 National Healthcare Group PolyclinicsSingapore; 6 Lee Kong Chian School of MedicineSingapore; 7 Westmead Applied Research Centre, The University of SydneyWestmeadAustralia; 8 Respiratory Health, The George Institute for Global HealthSydneyAustralia; 9 Centre for Research in Primary Health Care (CINAPS), Universidad Peruana Cayetano HerediaPeru; 10 Public Health Division, Pacific Community (SPC)SuvaFiji; 11 University of the PhilippinesManilaPhilippines; 12 Nuffield Department of Primary Care Health Sciences, University of OxfordUK; 13 Discipline of General Practice, School of Clinical Medicine, UNSWSydneyNSWAustralia

**Keywords:** Climate Change, Cardiovascular Health, Digital Primary Care, Digital Health Interventions, Scoping Review, Environmental Health

## Abstract

**Objectives:**

Climate change poses a major threat to cardiovascular health, particularly for those who are more vulnerable. Digital primary care (DPC) offers innovative ways to mitigate these effects by incorporating digital health interventions (DHIs) into primary care settings. This paper reviews recent work in integrating climate data with DPC systems to improve cardiovascular outcomes.

**Methods:**

We conducted a scoping review using PubMed to find studies from the past decade that examined how climate change affects cardiovascular disease (CVD) and primary care. We also included case studies from the International Medical Informatics Association Primary Care Informatics Working Group (IMIA PCI WG). We screened and selected studies based on predefined criteria that focused on climate change, CVD, and primary care integration, and data were extracted to identify research gaps and priorities.

**Results:**

We included eight studies from PubMed and seven international case studies from the IMIA PCI WG. Key studies have shown that cold weather, air pollution, and wildfire smoke can affect CVD outcomes. However, there is still a lack of evidence on effectively combining climate data with DPC tools in primary care settings to improve cardiovascular outcomes.

**Conclusions:**

DPC has the potential to improve cardiovascular health outcomes in the context of climate change. However, there is a need for more research and real-world evaluation of DPC tools. By integrating climate data with primary care systems, we can provide early warnings and targeted interventions, especially in low- and middle- income countries (LMICs). Future research should address these gaps to strengthen healthcare systems against the challenges posed by climate change.

## 1. Introduction

Climate change is a severe threat to health systems, altering disease patterns and medical services [ 1 ]. Climate health encompasses the impacts of climate change on health outcomes, including non-communicable diseases (NCDs). It includes a diverse array of issues, such as air quality deterioration, extreme temperature variations, heat stress, cold spells, and disease outbreaks due to changes in climatic conditions [ 2 ]. Among NCDs, cardiovascular disease (CVD) is one of the main causes of mortality worldwide and is strongly associated with environmental factors, including climate change, exposure to smoke, extreme weather conditions, and environmental pollutants [ 3 , 4 ]. Despite advancements in preventive and therapeutic measures, there is significant uncertainty regarding how environmental and climatic factors contribute to the development or worsening of CVD. This uncertainty highlights the need for thorough reviews to understand these connections better and guide the development of effective strategies for intervention. The burden of CVDs is rising continuously, particularly in low- and middle-income countries (LMICs), where >75% of global CVD-related cases occur [ 4 ]. Recent studies have shown that environmental factors have an increased impact on CVD mortality and morbidity, particularly in populations exposed to extreme environmental pollution or climate change. The rising incidence of CVD, obesity, and metabolic syndrome is linked to environmental and climatic factors, urbanisation, and lifestyle changes [ 1 , 4 – 6 ].

Digital primary care (DPC) can be defined as the systematic integration of digital technologies in primary care to deliver integrated care to prevent, diagnose, treat, and monitor health conditions. DPC is the discipline of knowledge and practice of adopting digital tools that help improve health in primary care, from inception to implementation. As climate change impacts health, DPC uses a OneHealth approach that can help tackle various chronic conditions, such as asthma and CVD, that are influenced by public health, agricultural, veterinary and environmental factors [ 11 , 82 ]. DPC involves digital health interventions (DHIs), wearable devices, electronic health records (EHRs), telehealth, and mobile health applications that facilitate timely monitoring and management of patients’ conditions. For instance, the Consumer Navigation of Electronic Cardiovascular Tools (CONNECT) trial showed that a web-based application linked to EHRs improved adherence to medication among high-risk patients with CVD in primary care by providing customisable reminders and feedback [ 7 ]. There’s an emerging need to understand how DHIs can be integrated into primary care, to improve patient care by preventing, diagnosing, treating, and monitoring health issues more effectively.

Primary care provides the best opportunity for designing, piloting, implementing, and evaluating DPC tools that link climate factors to human health and disease with or without a focus on body systems. Primary care settings provide unique opportunities to not only tailor these interventions at an individual level but also address the challenges at the population level. DPC connects the micro and macro aspects of health systems, especially in relation to climate sustainability. There are two major opportunities for DPC in addressing climate change. First, using informatics solutions can enhance care delivery by tackling common primary care issues like long wait times, access, and equity; while also looking at how these tools impact the environment and climate. For example, using virtual care more often can help cut down on greenhouse gas emissions, which is a step toward tackling climate change. Second, DPC tools can help people cope with the health effects of climate change. By educating folks on how extreme heat and poor air quality can make conditions like asthma and COPD worse, these tools give them the knowledge they need to take charge of their health proactively within their ecosystem.

Due to the community setting and access of both health and social care teams to primary care, the adoption of DPC tools can improve integration, improve economies of scale, and provide a holistic patient-centred approach to improve health outcomes. For example, targeted DHIs can be delivered to vulnerable populations in communities during extreme climatic events [ 8 ]. Timely remote monitoring and virtual consultations using telehealth for patients in remote regions enable access and health equity [ 9 ] as well as reduce the carbon footprint. The COVID-19 pandemic has demonstrated the importance of swiftly adopting DHIs in primary care and beyond to improve health outcomes [ 10 , 11 ].

DPC tools can provide targeted interventions, such as health education, monitoring of dietary and fitness patterns, and early alerts in the context of climate change. A healthy lifestyle can be promoted through wearable technologies by tracking the fitness activities [ 9 , 12 , 13 ]. Several DPC tools exist that promote healthy eating habits customised specifically for users [ 14 ]. These tools can also be used to enhance social support networks necessary to maintain resilience during extreme climatic events and natural disasters. Anxiety and stress due to climatic events cannot be ignored in primary care. However, many DPC interventions or tools have not yet been developed or assessed in the context of climate change. Integration and implementation of DPC is lagging, although significant application has been observed in secondary care, including the use of robotic technology, remote monitoring of cardiac rhythm, and function of biventricular pacing devices. Primary healthcare is at the forefront of primary prevention, playing a key role in detecting CVD at an early stage of the disease trajectory and assessing the risk of environmental risk factors, including climate change, on CVD health. AI-driven DPC can predict early CVD and suggest management to improve disease outcomes.

This review aims to identify the clinical and research priorities and gaps in DPC systems to better understand and manage the health effects of extreme climate and environmental changes. It focuses on how DPC can help monitor, prevent, and address the health impacts of climate change, particularly cardiovascular health. By systematically embedding climate and environmental change considerations into DPC systems, we can make healthcare more responsive to the health challenges posed by climate change. This scoping literature review and illustrative country examples using CVD as the primary tracer condition (but not excluding other body systems) to demonstrate the potential.

## 2. Methods

A scoping review was undertaken, followed by an expert consensus on the key findings [ 15 , 16 ]. Our emphasis was on identifying research gaps and priority areas and providing a comprehensive summary of literature to identify key concepts, gaps and categories of research that have been conducted [ 17 ].

### 2.1. Identification of evidence

We performed a literature search using PubMed. We identified studies published in the last 10 years by using a combination of keywords based on climate change, CVD and primary care. The search strategy and syntax are described in the Supplementary file. We then supplemented the review findings with country contributions from the International Medical Informatics Association Primary Care Informatics Working Group (IMIA PCI WG) members. These contributions include studies and reports specific to member countries, particularly case studies that investigated the effects of climate and environmental calamities on CVD.

### 2.2. Screening and selection criteria

The search results from PubMed were exported, and duplicate records systematically removed. We included papers that stated that the adult population was affected by cardiovascular disease due to climatic effects; studies of paediatric populations were excluded [ 18 ]. We only focused on studies that addressed how environmental or climatic factors contribute to the development or exacerbation of CVD. These factors may encompass a wide range of influences, which include air pollution, extreme temperatures, natural disasters and other climate-related conditions.[ 1 ] The outcomes included cardiovascular events across the primary, secondary, and tertiary healthcare settings. These studies have focused on those that are directly or indirectly influenced by climatic conditions. Two independent reviewers screened titles and abstracts to assess the relevance of the studies. All studies were thoroughly evaluated against predefined inclusion and exclusion criteria. In cases of conflict, consensus was reached through discussion with the co-authors. Studies that were deemed eligible were selected for data extraction. The final inclusion criteria were studies that: i) incorporated the use of electronic health records (EHRs), and ii) integrated climate data with CVD-related data from primary care, including community health and general practice settings.

### 2.3. Data synthesis

Data were extracted from eligible studies and case studies nominated by co-authors using standardised templates. The data extraction template included information such as the study title, authors’ names, year of publication, study region (city, country), study period, study design, primary care data source, secondary care data source, climate data source, population size, climate exposure, digital intervention, control, outcome variables, key findings, limitations, and priority areas. The extracted data were then cross-checked and discussed to reach a consensus. The extracted data and templates are shown in the Supplementary File.

## 3. Results

Eight studies [ 19 – 26 ] were found to be eligible ( Figure 1 ). Additionally, seven case studies were identified. These studies collectively highlight the role of primary care in delivering cardiovascular care in the context of climate health, particularly during extreme air pollution, weather conditions, and events.

**Figure 1:**
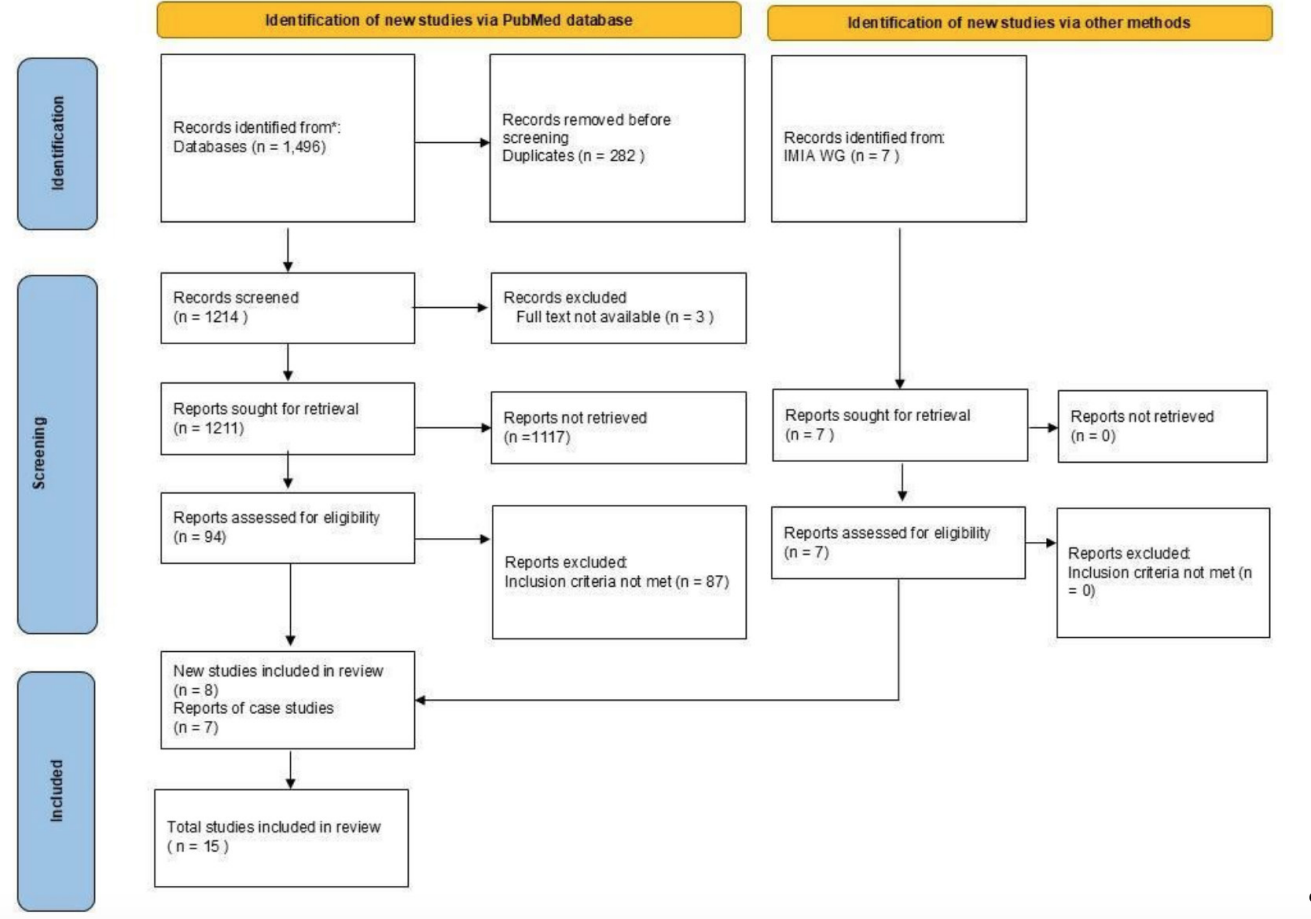
. Summary of eligible studies included in the synthesis.

A study in Spain focused on the impact of cold spells on CVD hospitalisations, in which demographic and other risk factor data were obtained from primary care [ 19 ]. The effects of suboptimal temperatures, particularly moderate cold, on rural populations, with cold temperatures contributing to delayed cardiovascular effects and an increase in primary care visits, were highlighted in a study conducted in China [ 20 ]. In another study from the United Kingdom, association between long-term exposure to particulate matter (PM10) and nitrogen dioxide with the development of CVD events identified using primary care visits, hospital admissions and death certificates particularly in more affluent areas [ 21 ]. Wildfire smoke exposure during the 2007 San Diego wildfires in the United States of America (USA) led to increased respiratory diagnoses, particularly among vulnerable paediatric populations and individuals with asthma, with persistent healthcare utilisation even after the initial exposure period [ 22 ]. Similarly, in British Columbia, Canada exposure to wildfire smoke, measured by PM10, was associated with increased primary care visits, hospital visits, and admissions, although no significant cardiovascular effects were observed [ 23 ]. During the 1999 Hoopa Valley wildfires in the USA, significant associations were found between PM10 and increased visits to regional primary care medical centres for asthma, coronary artery disease, and headaches [ 24 ]. The 2011 floods in Quebec (Canada) resulted in a notable increase in the CVD burden with an increased number of emergency department visits, outpatient visits, and community physician visits [ 25 ]. A Danish nurse cohort study focuses on the association between long-term exposure to air pollution and CVD events in primary and secondary care settings [ 26 ].

### 3.1. Priority Areas and Research Gaps

Our findings suggest that there is limited evidence regarding primary care interventions. Most of the studies used data collected from primary care facilities to investigate associations of CVD outcomes or service utilisation in relation to climate change, but did not deliver an intervention. In one study, although primary care data linked with hospitalisations were available, the focus was on CVD-related hospitalisations not clinical outcomes [ 19 ]. We believe that it is important to utilise and integrate primary care outcomes where appropriate. Another study which focused on impacts of cold temperature on rural population suggested the need to focus more on rural health. In the study by Zhao *et al.* [ 20 ], the focus is on mitigating long-term exposure to particulate matter to reduce the risk of cardiovascular events. Natural disasters, such as wildfires, can lead to an increase in respiratory-related healthcare visits, highlighting the need to mitigate these issues to reduce the incidence of respiratory-related diseases [ 21 ]. Vanasse *et al.* [ 25 ] found that due to floods, there is a 25%-27% increase in acute CVD and 69% in individuals with pre-existing CVD. This highlights a potential but inconclusive link between flooding and CVD health. The study by Cramer *et al.* [ 26 ] on the Danish nurse cohort investigates the impact of long-term exposure to particulate matter on fatal CVD outcomes, particularly myocardial infarction (MI), while also addressing road traffic noise as a potential confounding factor. It highlights the role of occupational exposure and emphasises the impact of air pollution on CVD risk.

Higher physical activity levels are associated with improved health outcomes in patients with cardiovascular and metabolic diseases. Previous studies have indicated that climate and environmental factors, such as temperature, climate events, availability of cycling infrastructure, and urban design, significantly influence physical activity levels [ 27 – 29 ]. For instance, research has shown that cities with better cycling infrastructure tend to have higher cycling participation rates, which in turn can lead to improved public health outcomes [ 30 ]. Moreover, studies have highlighted disparities in physical activity levels based on socioeconomic status, age, and gender [ 31 – 33 ]. However, there is limited evidence on the relationship between climate, social infrastructure, CVD health outcomes, and digital health interventions in primary care. Wearable devices are often used to track physical activity.

Although there are other studies that [ 27 – 29 ] explored the associations between physical activity patterns, such as cycling, and CVD outcomes, we did not find a study that integrated these data with climate data in the form of a DHI. Real-time integration of physical activity data obtained through a digital health application with climate, health services, and administrative datasets can provide a holistic view of an individual’s health and an opportunity to intervene at various stages. The integration of these heterogeneous data sources can be challenging and a systematic approach and framework is required.

Digital health has significant potential for lowering greenhouse gas emissions by reducing the need for travel, expediting healthcare processes, and improving overall operational efficiency. However, its wider adoption may also result in increased energy consumption, particularly because of its dependency on data centres, cloud computing, and digital infrastructure [ 2 ]. Our review did not find any studies addressing this issue in the context of DPC or CVD.

The following identified research gaps are highlighted in both high-income countries and LMICs. More research is needed with a focus on integrating climate and CVD data to overcome this gap, which, in turn, will allow the development and delivery of contextually sensitive interventions to specific populations in mind such as culturally and linguistically diverse (CALD) populations. Anxiety and stress due to climate change have not been widely studied, highlighting the need to integrate mental health support related to the climate within primary care. These findings on the need to focus on LMICs, role of digital health, primary care and primary care informatics were also highlighted in two other reviews [ 5 , 6 ].

### 3.2. Case Studies

#### Australia – electronic Practice Based Research Network (ePBRN)

One of the objectives of the ePBRN program is to investigate the complex association between climate factors, such as temperature variations, and cardiovascular disease (CVD) health outcomes across diverse demographic groups in Southwest Sydney (SWS) using routinely collected general practice EHR data. This interdisciplinary approach provides valuable insights into the role of climate and lifestyle in shaping health outcomes, ultimately informing targeted interventions and policies to promote sustainable and climate-resilient urban living. The University of New South Wales (UNSW) electronic Practice Based Research Network (ePBRN) program was established in 2010 in SWS to undertake primary care clinical and health services research. The ePBRN program extracts and links EHRs data from consented General Practices (GPs) and local health services[ 34 , 35 ]. The SWS region and Australia in general suffers regularly from extreme climate events such as floods, cyclones, bushfires and heat waves. ePBRN is a representative sample of SWS individuals who use health services. Individuals in this region often have CALD backgrounds and face significant socioeconomic challenges. Integration with local climate data allows researchers to analyse how these patterns correlate with CVD health outcomes in the ePBRN dataset. Several climate based indicators can be devised using the geocoded data in ePBRN. For example, meteorological data such as temperatures and particulate matter information is used to understand the service utilisation through continuity of care indices during extreme weather events. However, challenges exist in integrating these heterogeneous data sources, which can generate evidence beyond CVD and climate health, pertaining to urban, civil, or city planning.

#### Bangladesh

Bangladesh is one of the most densely populated countries in the world. It is affected by floods, droughts, cyclones, tornadoes, and extreme weather conditions, including heat waves, sandstorms, earthquakes, and political conflicts. Recently, there has been an influx of over 1 million refugees from Myanmar in the coastal region of Cox’s Bazar, which has overwhelmed the fragile health and social care system. People live in makeshift tents without proper sanitation or a safe water supply. The country is struggling to meet the necessities of the migrant population, and this is creating conflicts with the local communities. Furthermore, due to adverse climate changes, including landslides and river erosion, people are moving en masse to big cities, putting additional pressure on health and social care infrastructures. In addition to natural disasters such as floods, storms, and cyclones, the country’s reliance on fossil fuels for generating energy, emissions from garment factories, and air pollution due to toxic greenhouse gas emissions from automobiles is adding fire to the fuel of climate change.

Between 2000 and 2024, overall population density increased from 992 per square kilometre to 1350. Population density is considerably higher in urban than rural areas, around 755 per square kilometre. The World Risk Index 2023 ranked the country as the ninth most vulnerable to climate disasters [ 36 ]. It is estimated that by 2050, owing to rising sea levels, 30% of the country’s agricultural land will be lost [ 37 ]. Global warming is accelerating the melting of Himalayan glaciers, which inundate the river system, causing flooding, river erosion, and landslides. Additionally, human-created pollution, including the indiscriminate dumping of industrial waste, non-biodegradable polyethylene, and plastics in rivers and canals, causes the landfill of rivers and obstructs water flow. Severe flooding and waterlogging affect 55% of the country’s total area. The World Bank estimate suggests that the mean temperatures will increase between 1.4°C and 2.4°C by 2050 and 2100 [ 38 ]. People migrate from villages to cities because of river erosion, landslides, and loss of livelihood every year. Climate migrants live in overcrowded slums with poor access to water, food, sanitation, and hygiene, resulting in poor health outcomes, food insecurity, and suffering from a diverse range of communicable and non-communicable diseases, including cardiovascular diseases.

EHR in Bangladesh is at its infancy. Moreover, there is no structured and universal primary health care (PHC) service available at the point of need. In some parts of the country there are community clinics, which has generally improved access to PHC [ 79 ]. Most of the clinics have been funded by the USAID. Due to the recent change in funding of USAID, it is likely that these clinics will not continue. Likewise, there is no requirement that patients attending those clinics continue to receive care from the same. Patients may choose to see a private physician or move to another clinic resulting in multiple health records. Health records are paper held and, in most instances, not available in subsequent consultations. Similarly, although the country faces enormous challenges in climate changes, the impact of it on health is still not recognised by the policy makers and healthcare professionals [ 80 ]. Therefore, there is a lot of opportunity to raise awareness and incorporate climate and EHR in Bangladesh.

#### China - Shanghai’s health risk forecasting system

By leveraging data-driven approaches, Shanghai developed a health risk forecasting system to promote primary care-based climate resilience and address the effects of drastic climate events, such as floods, earthquakes, cold spells, and heatwaves [ 39 ]. The key features of the system are real-time monitoring, focus on vulnerable populations, public awareness and communication, and collaboration across sectors. This system integrates meteorological, environmental, and health data to predict and mitigate health risks associated with extreme weather events, pollution, and other climate-induced factors, and issues early alerts and warnings to vulnerable populations, such as the elderly and those with chronic diseases.

The integrated service for chronic disease management delivered through this system reduced consultations and costs by 17.6% and 2.5%, respectively. The implementation of targeted alerts and cooling centres has also reduced heat-related consultations. This model of care demonstrated a significant impact on improving health outcomes, which substantially reduced hospital admissions by providing early warnings related to climate stressors and events such as heat waves, pollen, and floods. Adjusting to changing climatic conditions enables health systems to be more resilient to extreme weather and environmental hazards. This has several economic benefits because it lowers the expenses associated with emergency responses and healthcare during climate-related crises.

#### Canada – National Adaptation Strategy

A pressing issue in Canada, as well as elsewhere, is the need for an integrated primary care delivery system. Canada’s primary care system suffers from multiple issues including access issues, a lack of team based care delivery, and poor integration of virtual care and other aspects of primary care delivery [ 40 ]. Climate can impact human health in direct and indirect ways [ 41 ]. Direct impacts include health impacts from climate events such as fires while indirect impacts can include health system issues such as access or health human resource issues. Extreme heat events are becoming a common occurrence in Canada and these events can greatly impact human health [ 42 ]. Respiratory related illness [ 43 ] and CVD [ 44 ] are greatly impacted by climate change.

Canada has developed the National Adaptation Strategy to help understand, prepare and develop proactive solutions to mitigate the impact of climate change. The strategy has three main pillars: Knowledge and understanding, tools and resources, and governance and leadership. However, a challenge is that macro level government frameworks are removed from the design and delivery of health services as Canada lacks an integrated framework to integrate human and environmental health. This lack of integration results in two issues. First, we lack the ability to understand and evaluate the environmental impact of digital health tools across micro, meso and macro systems [ 45 ]. Second, it makes it difficult to integrate climate related data into micro level care planning and delivery for individual health management.

Given the increasing frequency in Canada of climate events such as extreme fires, a proactive approach would allow us to better help individuals manage the impact of climate change on individual human health while also being mindful of macro level impacts of health service delivery on climate systems. Weather networks and Government agencies monitor air quality ratings, but they are not integrated with health data to allow for real time health management in response to changing air quality. DPC solutions could assist people with respiratory or CVD issues to enable proactive management of their health during periods of poor air quality due to fires or other air quality related climate events.

#### Indonesia - SatuSehat National Data Platform

Indonesia is undergoing both an economic and demographic transition. Economically there’s a shift towards industrialization and services while still having primary industries such as mining and logging being a substantial contributor to the economy. Whereas demographically Indonesia is expected to reach its peak population in two decades (by 2045) with most of the population being elderly by the end of the century. Both trends combined have resulted in changes where lifestyle factors, air pollution and occupational risks are now the top 10 risk factors for morbidity, and CVD being the top cause of death [ 66 ]. The WHO also predicts that climate related changes especially relating to rising temperatures would be a significant contributor of death by 2030 ( Figure 2 ). This increase in death will be most apparent in those aged 65 years and older with previous studies reporting heat related stress significantly increasing the risk of CVD mortality especially in those old and with pre-existing disease [ 67 ].

**Figure 2:**
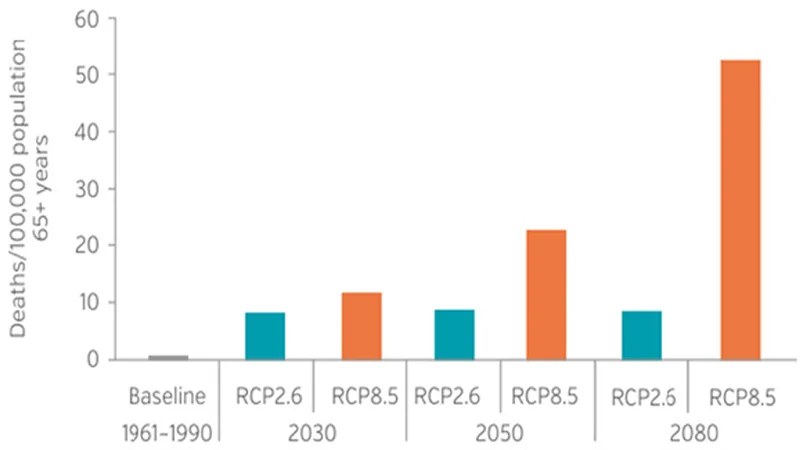
. Heat-related mortality in Indonesia in individuals 65 years or over. RCP - Representative Concentration Pathway. Source: https://cdn.who.int/media/docs/default-source/searo/wsh-och-searo/ino-c-h-prof.pdf?sfvrsn=57b5ca43_2

While CVD is top of the list, communicable diseases such as diarrhea, further worsened by the increasing severity and commonality of natural disasters (e.g., flooding) due to climate change also remain in the top 10 [ 68 ]. For example, heat stress and abundant rainfall had raised dengue fever cases by 227% in Bali-Nusa Tenggara, and malaria cases in Papua by 66% while pneumonia cases had conversely increased by 96% due to temperature falls and limited rainfall in Maluku [ 69 ].

A lack of infrastructure and coordination across agencies remain one of the barriers to an effective response to climate change [ 68 ]. Post COVID-19, Indonesia had transitioned its mobile contact tracing platform, Peduli Lindungi, into a citizen’s health app under the national integrated health data exchange ecosystem called Satu Sehat (One Health in English) [ 70 ]. The platform acts both as a personal health record where users can book vaccinations and receive vouchers to undertake free annual health checks, but also as an ecosystem acts as a national data lake where all accredited health facilities (including primary care clinics) in Indonesia with an electronic medical record system are obliged to push data regarding healthcare visits as mandated by the 2023 Indonesian Healthcare Act No 17 using the FHIR standard.

In its first year of operation, the Ministry of Health has provided some evidence that data flows are usable for analysis, especially for NCDs, although there remains work to validate the data quality and link it to other siloed systems such as data regarding air quality, weather, and heat currently housed under disparate systems managed by meteorological and environmental agencies. Linking these disparate systems and having them accessible in primary care clinics starting with the >10,000 government owned community health centres (Puskesmas) which are tasked with population health management would significantly increase Indonesia’s health system resilience in the face of climate change and support generation of new evidence to bridge many of the gaps in our understanding on the links between climate change and health.

#### Philippines

An archipelago along the Ring of Fire, the Philippines experiences diverse extreme weather, directly and indirectly impacting health. Typhoons, floods, volcanic eruptions, and extreme heat often coincide with water-related diseases like leptospirosis and diarrhea. Ecological shifts accompanying these weather changes can increase vector activity and infections such as malaria and dengue fever. The poor air quality resulting from climate change directly affects respiratory and cardiovascular systems. Aside from the physical effects of climate change on health, recent data shows its effect on Filipinos’ mental health as well [ 63 ]. Climate anxiety or eco-anxiety, while not a clinical disorder, is a psychological response to severe environmental conditions. Recognizing this climate-health link, the Philippine government prioritized research funding on climate change’s health impacts. A stakeholder analysis facilitated government collaboration with research and other entities to generate studies on climate change and health [ 64 , 65 ].

An emerging opportunity is the new 8-point action agenda which explicitly states the use of technology for more efficient healthcare delivery [ 81 ]. To prioritize, the Philippine Department of Health identified eight priority areas one of which included hypertension. For the period of 2023 to 2028, government will invest in eight tier1 projects such as electronic medical records in all healthcare facilities and a national health data repository (aka, electronic health record) for all citizens of the country at all levels of the healthcare system starting from the community.

#### Singapore – Green Plan

Singapore is in Southeast Asia, located at the southern tip of the Malay Peninsula, 137 kilometres (85 miles) north of the equator. High rainfall and hot weather characterize the round-the-year climate of Singapore, and its climate is influenced by major climatic phenomena, such as the El Niño–Southern Oscillation (ENSO). In the last decade, Singapore has experienced global warming at twice the global average, and this has been accompanied by an increase in rainfall [ 46 ].

The increase in temperature and humidity has contributed to an increase in infections, such as dengue (a mosquito-borne disease) [ 47 – 49 ], Salmonellosis [ 50 ] and diarrheal disease [ 51 ]. Hand, foot and mouth disease, largely a paediatric disease caused by the human enterovirus or coxsackievirus, has also increased in association with temperature and humidity increase [ 52 , 53 ]. People in Singapore are physiologically and behaviourally adapted to the tropical climate in their daily life, and severe heat-related illnesses such as heat stroke, heat exhaustion and heat syncope usually occur in association with vigorous physical activity and in overseas visitors [ 54 ].

The Singapore Green Plan, Singapore’s first environment blueprint, was first released in 1992, to ensure that Singapore’s economic growth model did not compromise its environment [ 55 ]. With increasing concern over global environmental issues, an inter-ministerial committee on climate change was established in 2007 to enhance whole-of-government coordination on climate change policies. The Singapore Green Plan 2030, launched in 2021, was expanded to include a whole-of-nation movement to advance Singapore’s national agenda on sustainable development. In addition to the Ministry of Sustainability and the Environment (MSE), it is also spearheaded by four other ministries - Trade and Industry (MTI), Transport (MOT), National Development, and Education (MOE). There are five main areas of focus- city in nature, energy reset, sustainable living, green economy and resilient future.

In the healthcare setting, a sustainability office within the Ministry of Health Holdings has been charged with coordinating and supercharging efforts across the public healthcare sector, including primary care. The Agency for Logistics and Procurement Services, a public healthcare supply chain agency, recently unveiled its green procurement guidelines to improve energy and water efficiency and to reduce waste in public healthcare delivery [ 56 ]. There is increasing focus on how to decarbonise healthcare in Singapore, with green initiatives operationalised in various healthcare clusters [ 57 – 60 ], dedicated educational programs focusing on teaching sustainability skills [ 61 ] and research centres to address climate change issues [ 62 ].

## 4. Discussion

### 4.1. Underutilisation of DPC Tools

Digital health interventions (DHI) play a pivotal role in improving the outcomes of cardiovascular disease (CVD). DHI can significantly reduce CVD events, mortality, and hospitalisations compared to usual care settings, as interventions can be made easily accessible through various digital and mobile technologies. There are various mobile applications to detect early symptoms and provide treatment recommendations for CVD and other medical conditions [ 71 ]. For example, the DETAK-C mobile application [ 72 ] was designed to facilitate early detection and treatment of acute coronary syndrome (ACS) in a community setting. The application is actively used by over 1,000 individuals in the community of Malang, Indonesia. The key features include triage, service navigation and educational resources. The DPC tool [ 73 ] also tracks the associated symptoms and outcomes. Multiple studies not meeting the inclusion criteria of our study investigated the associations between hospitalisations, outpatient hospital visits, and climate events. For example, dust storms in Taiwan were linked to increased CVD admissions, particularly on the day after the storm, although the findings were not statistically significant [ 74 ]. In another study, short-term increases in PM2.5 were linked to a higher risk of atrial fibrillation episodes, with particular emphasis on low-pollution environments [ 75 ]. Data on atrial fibrillation were extracted and analysed in outpatient clinics. Data from devices such as this provide an opportunity to integrate information in real-time with climate data to deliver person-centred alerts or warnings in the form of a DHI. However, we did not find a study that integrated CVD and climate health data in either high-income countries or LMICs.

The underutilisation of DPC tools and broadly DHIs could be due to various reasons, such as a lack of capacity and the realisation of digital capabilities. Technical and implementation challenges, such as integration, interoperability standards, and ethical and privacy concerns, could also have a role and cannot be overlooked. For example, interoperability standards-related challenges were observed in the ePBRN case study. A mature taxonomy or ontology has not been identified to integrate and store climate data with general practice and hospital data capable of capturing knowledge and associations. This limits the digital phenotyping and research hypothesis generation capabilities of the program. A possible solution is to integrate environmental considerations into an organisation’s digital health technology strategy and implementation roadmaps, especially in LMICs. This approach will not only encourage the development, evaluation, and adoption of DHIs that are interoperable, but also leverage the emerging capabilities of mobile phones to capture and integrate climate and other related data seamlessly [ 2 ]. For instance, this type of integration will allow the DETAK application to investigate the associations and ability to provide early alerts to Indonesian users based on adverse climatic conditions which are common, similar to Shanghai’s health risk forecasting system but more personalized [ 39 ].

### 4.2. Ethical and Privacy Challenges

The integration of health data from diverse sources, particularly in broader contexts, such as climate health, presents significant privacy and ethical challenges. Key challenges include balancing technical innovations in digital health with privacy, ensuring data integrity, and maintaining trust in health care systems. Various regulatory and legal frameworks established worldwide provide guidelines on how to address these issues. These include the Health Insurance Portability and Accountability Act (HIPAA) from the United States of America, Privacy Act of 1988 from Australia, and European Union’s General Data Protection Regulation (GDPR). Although these frameworks provide well-established guidelines to preserve patients’ privacy, they are often generic and do not highlight ethical and privacy risks in the context of climate events. Sensitive health information captured in sources such as EHRs need to be safeguarded before and after linking with other data streams such as wearable data [ 76 ].

Privacy-preserving techniques, such as differential privacy, deidentification, and surrogation, have been developed for data integration. Liu *et al.* [ 67 ] presented an automated deidentification pipeline for unstructured EHRs, which incorporates both rule-based techniques and transformer models. Similarly, right techniques can be incorporated for other data types, such as wearable data, before linking them with EHR or climate data. The practical implementation of these techniques remains challenging with rapidly evolving technologies such as generative artificial intelligence. Another challenge is the management of patient consent for complex data integration [ 77 ]. A patient might consent to integrate or link their data to a specific data stream and might revoke later. Implementation and governance challenges in monitoring and maintaining dynamic consent which can be more complex when multiple data streams exist, wherein the data ownership and privacy legislations might vary between each data stream [ 78 ]. LMICswith limited access to resources, infrastructure, and facilities are vulnerable to these challenges. However, incorporating context-aware privacy strategies into a new tailor-made or adopted data integration framework can assist in addressing these challenges [ 76 ].

## 5. Conclusion

Our review highlights that there is a global need to connect human and environmental health strategies to tackle the rising issue of CVD as climate change worsens. Currently a global challenge is that environmental and human health systems are isolated entities and that limits our ability to bridge the two systems in pursuit of shared societal goals. We found some important gaps in how primary care deals with climate-related issues. Our case studies showed that climate events can directly and indirectly affect CVD outcomes. We also found some exciting solutions, like real-time health risk forecasting and digital health platforms that use climate data to improve patient care. Our review of DPC tools points out their potential to connect local health data with wider climate information, but we also found there are not enough systems that allow smooth data sharing. To move towards healthcare that can withstand and respond robustly to climate challenges, the primary care and health informatics community needs to embrace the OneHealth approach. This means developing shared multidisciplinary frameworks and effective data-sharing methods across professions and health systems. Future research should focus on integrated models, especially in low- and middle-income countries, to ensure that we have proactive and context-specific strategies to reduce climate-related cardiovascular risks.

## Primary Care Informatics Work Group: Appendix

**Table:** 

**Search strategy ** **Database: PubMed ** **Number of studies identified: 1,496**
((“climate change” OR “greenhouse effect” OR “global warming” OR “extreme weather” OR “extreme hot weather” OR “hot temperature” OR “floods” OR “sea level rise” OR “natural disaster” OR “extreme cold weather” OR “hurricane” OR “cyclonic storms” OR “cyclonic storm” OR “cyclone” OR “monsoon” OR “salt water intrusion” OR “tropical storm” OR “heat wave” OR “wildfires” OR “forest fire” OR “fires” OR “fire season” OR “air pollution” OR “smoke” OR “smoke event” OR “migration” OR “temperature variability” OR “cold spell” OR “environmental health” OR “climate-related health effects” OR “climate-sensitive diseases” OR “climate resilience” OR “particulate matter”) AND (“cardiovascular disease” OR “heart disease” OR “coronary artery disease” OR “heart failure” OR “congestive heart failure” OR “acute heart failure” OR “systolic heart failure” OR “diastolic heart failure” OR “heart arrest” OR “ischemic heart disease” OR “heart muscle ischemia” OR “myocardial infarction” OR “heart infarction” OR “cardiac arrhythmia” OR “heart arrhythmia” OR “cerebrovascular accident” OR “stroke” OR “cerebrovascular disorders” OR “cerebrovascular disease” OR “cardiovascular outcomes” OR “cardiovascular risk” OR “cardiovascular health” OR “cardiovascular events” OR “cardiovascular morbidity” OR “cardiovascular mortality”) AND (“primary care” OR “family physician” OR “primary health care” OR “general practice” OR “general practitioners” OR “nurse practitioners” OR “community healthcare provider” OR “physician” OR “primary healthcare” OR “primary care physician” OR “primary care provider” OR “family medicine” OR “family practice” OR “community health” OR “community health service” OR “general medical practice” OR “preventive health service” OR “health services research” OR “primary care services” OR “primary care intervention” OR “community health nursing” OR “physicians primary care” OR “patient-centered care” OR “home care service”))
